#  BlindSpot facilitates unbiased data analysis via file renaming

**DOI:** 10.17912/micropub.biology.002154

**Published:** 2026-10-26

**Authors:** Jenna S Vesey, Amy B Liu, Siddharth K Sankaranarayanan, Amy S Maddox, Linnea C Wethekam

**Affiliations:** 1 Department of Biology, UNC-Chapel Hill, Chapel Hill, NC, US

## Abstract

Bias and subjectivity are to be avoided in scientific research since they can lead to incorrect data interpretation. Methods that enhance the rigor and reproducibility of biomedical research include filename blinding. To overcome the limitations of currently available blinding programs, we developed *BlindSpot,* a publicly available, user-friendly tool for filename renaming. *BlindSpot* is compatible with macOS and Windows, supports both a graphical user interface (GUI) and a command-line interface (CLI), and has features to customize blinding and safeguard data identity.

**Figure 1. Overview of the BlindSpot pipeline for reproducible filename blinding and restoration:** A - H) blinding pathway (green arrows). I - M) unblinding pipeline (purple arrows). A) User identifies target folder for blinding. B) User accesses the graphical user interface (GUI), selects specified inputs, designates the target folder, specifies the file type, and makes optional selections. C) Once blinding is complete a pop-up shows a summary of the blinding. D) Example output where with no image duplication or file movement. E) Example output where blinded files were duplicated and moved to a new folder. F) Example including subdirectories with the blinded files having been moved to a new folder. G) Layout of the Blinding_key.csv for data identity restoration. H) Layout of the _blinding_log.csv showing tracked and recorded actions. I) Example folder containing blinded data. J) Unblinding function tab. K) GUI popup after unblinding is complete. L) Output folder after running the unblinding code. M) Printed output _unblinding_log.csv.

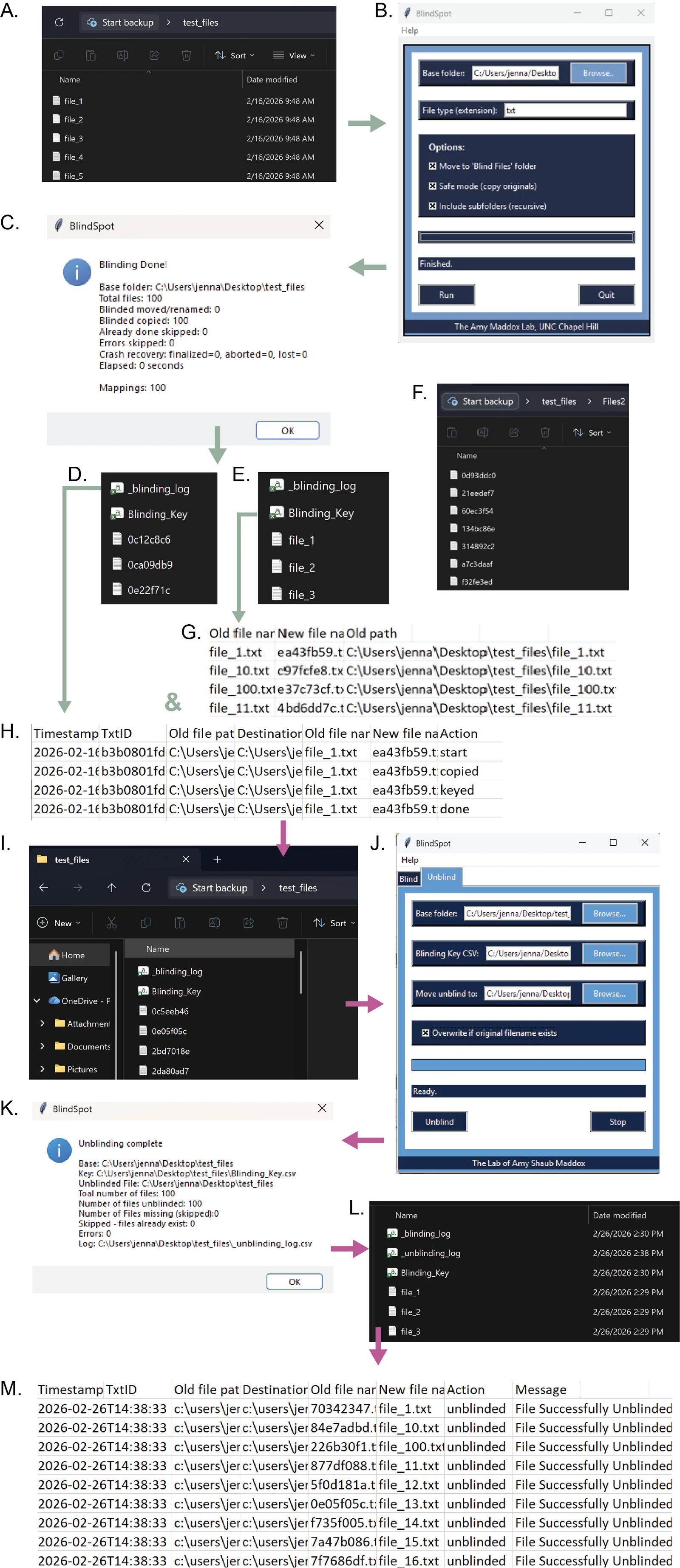



## Description

Scientists strive for objectivity to ensure the integrity of results, yet a “reproducibility crisis” exists (Bespalov et al., 2020; Baker, 2016). As few as 39% of published studies have been reproduced (Youyou, W., Yang, Y., & Uzzi, B. 2023). One basis of irreproducibility is error introduced by investigator bias (Bello et al., 2014; Jost and Waters 2019). Blinded analysis reduces bias and increases reproducibility (Baker, 2016; Jost and Waters 2019). However, blinding is not consistently reported in the analysis of microscopy images and other datasets, in part because it can be tedious (Begley and Ioannidis 2015; Bello et al. 2014; Bespalov et al. 2020; Karp et al. 2022; Jost and Waters 2019). While most blinding programs are specific to file format, Blinder and ImageJ’s blinding plugin can be used for multiple file types (Cothren et al., 2018; Jaiswal & Lorenz, 2019; Schneider et al., 2012). However, both temporarily rename files within a user interface – either an independent GUI or ImageJ – limiting the portability of the files for image analysis. We developed *BlindSpot* to stably blind file names in a flexible and accessible manner.

In designing *BlindSpot,* we prioritized crash safety and accessibility for non-programmers. *BlindSpot* not only accomplishes filename randomization, but also provides crash-safe bookkeeping that guarantees each file is renamed exactly once. Importantly, *Blindspot* restores a file’s original name after analysis is complete.

To begin, users open *BlindSpot’s* graphical user interface (GUI) on their device and identify the desired folder for blinding and select the required features ( Figure 1A, B). The program first confirms files of the specified type are present, and if directed, also searches all subfolders ( Figure 1A, B). To blind each file, the program selects a file with the correct extension and verifies that it has not already been blinded, using _blinding_log.csv ( Figure 1D- F). If the user elects for the blinded file to be a copy of the original file, the original file is retained and thus protected. If the user does not select this option, a warning message prompts the user to confirm the intention to overwrite filenames. Files are then renamed randomly using the final 8 characters from the universally unique identifier (UUID) package (Leach et al., 2005). Progress of the blinding is logged into _blinding_log.csv throughout the process; Blinding_key.csv is produced after all files are blinded ( Figure 1G ). Within _blinding_log.csv, each step of the blinding process (Starting, Keyed, Pending Skipped, Error, Copied, Moved, and Done) is logged with a time stamp ( Figure 1H ). Blinding_key.csv contains the original file name, the corresponding blinded file name, and the directory path of the original file ( Figure 1G ). If a crash occurs during the blinding of a set of files, upon re-initiation, the program will pick up where it left off, such that each file is blinded once. A progress bar is visible in the GUI as blinding occurs.

To restore original filenames, *BlindSpot* uses blinding_key.csv, the location of the blinded files, and the user’s desired output location to run this program. First, all files represented in the key are identified by searching the designated directory and its subfolders ( Figure 1I, J). File names not found are ‘Skipped.’ Unblinded files are marked ‘Done.’ Finally, if all of the program’s required conditions are met, the file is unblinded and placed in the user-specified directory ( Figure 1L ). Users can choose to overwrite the original files with the blinded modified file ( Figure 1J ), though situations where multiple files would result in the same filename are not automatically resolved. Unblinding creates a log summary and greets the user with a random quote after the software is done with its run ( Figure 1L, M).

To incorporate *BlindSpot* tool into extended workflows, the ‘blind’ and ‘unblind’ functions can be added to python workflows with the use of PyPI packages and command line interface. Documentation on how to call and utilize *BlindSpot* in the command line is present in the Readme.md on GitHub. Directions for downloading the software is available in the GitHub page in: “BlindSpot Installation Instructions”. Future development will focus on integrating *BlindSpot* with popular imaging analysis software and improving compatibility with automated and computational pipelines.

## Methods

*BlindSpot* was written in Python version 3.14.2 using Visual Studio Code. It was formatted using the program package “black” to enable easy readability (Langa & Black contributors, 2026). *BlindSpot* was compiled with the use of bin for Windows and bash for Mac using PyInstaller (v 6.19.0) and tkinter via command line functionality ( *PyInstaller development team. PyInstaller (Version 6.19.0)).* The program uses the following natively installed python packages: os, csv, uuid, time, shutil, random, threading, pathlib, tkinter, datetime, webbrowser, and sys. The logo lil_timmy.png was drawn in MS paint (v Paint 11.2512.211.0, Microsoft Corporation) and animated with the use of Canva’s gif function.

To test the robustness of *BlindSpot,* pytest was used (pytest developers. Version 9.0.2), a Python package that allows functions to be tested independently. This program was run through stress tests to confirm that each of 32 elements of *BlindSpot* can function and recover amid any errors (see Blinder_test.py, Unblinder_test.py, Crash_log_test.py, and File_processing_test.py on GitHub). File_processing_test.py documents the testing of these three functions: file naming, file folder, and path cross-compatibility among operating systems. Fourteen functions test the integrity of the crash log. It checks that the software identifies files that have been processed, recovery in the case of a system crash, and that all user-selected options would be consistent across each file run. A test of nine features assessed integrity of the blinder function (Blinder_test.py). Each of these functions ensure that regardless of user settings, the program calls each function correctly and accurately for every file. Each of the twenty-five tests ran in under one minute, including trials with 1000 files, though duration of our tests reflected file size. The unblinding function was also tested to ensure that unblinded files would be properly matched with original files the corresponding name. Tests were performed on both Windows and MacOS. Web application and code can be accessed at this link: https://github.com/maddox-lab/BlindSpot.git . Detailed instructions on how to download BlindSpot can also be found in this folder labeled “BlindSpot Installation Instructions”.

**AI usage:** AI assistance (ChatGPT-5.3, OpenAI) was used to suggest debugging strategies and interpret error messages during development. No AI assistance was used to write or edit the manuscript. The pylint package helped identify errors for formatting and structure for further review (pylint contributors, n.d.). The Black formatter was used for code formatting (Langa, Ł., & contributors to Black, 2026). The code was tested under human scrutiny and checked against current literature regarding the code for accuracy of function and layout. All code functionality and design decisions were verified by the authors.

## Data Availability

Description: Zip folder of the GitHub repository containing both PC and MacOS BlindSpot Software and all accompanying documentation.. Resource Type: Software. DOI: https://doi.org/10.22002/5zfem-kkx10
